# Environmental Performance in EU Countries from the Perspective of Its Relation to Human and Economic Wellbeing

**DOI:** 10.3390/ijerph182312733

**Published:** 2021-12-02

**Authors:** Simona-Roxana Ulman, Costica Mihai, Cristina Cautisanu, Ioan-Sebastian Brumă, Oana Coca, Gavril Stefan

**Affiliations:** 1 CERNESIM Environmental Research Center, Institute of Interdisciplinary Research, “Alexandru Ioan Cuza” University of Iasi, 700505 Iasi, Romania; simona.ulman@uaic.ro; 2 Faculty of Economics and Business Administration, Alexandru Ioan Cuza University of Iasi, 700505 Iasi, Romania; ticu@uaic.ro; 3 Romanian Academy, “Gh. Zane” Institute for Economic and Social Research, 700481 Iasi, Romania; 4 Faculty of Agriculture, “Ion Ionescu de la Brad” Iasi University of Life Sciences, 700490 Iasi, Romania; stefang@uaiasi.ro

**Keywords:** environmental performance, human and economic wellbeing, EU member states, cluster, panel data models

## Abstract

The actual development challenges impose new criteria of national performance evaluation, the concept of wellbeing tending to be measured not just in terms of economic and social dimensions, but also vs. the environment. Accordingly, considering the national environmental performance among the EU countries in 2006–2019 period, we grouped them and concentrated on the clusters registering the highest and lowest levels, analyzing how the components of the human and economic dimensions influence it. Applying panel data models, our main results emphasized that, firstly, for the countries with a better environmental performance, sufficient drinking water, safe sanitation, education, gender equality, and good governance were significant; in the countries with the lowest levels of environmental wellbeing, sufficient food, sufficient to drink, education, and income distribution were insignificant, while the remaining components were relevant. Secondly, in both groups of countries, organic farming and public debt were significant; nevertheless, differences were observed for genuine savings and employment, for which the peculiarities of economic activities seemed to be materialized as different influences upon environmental wellbeing. Our study draws alarm signals regarding the development patterns applied in the EU, seeming to have results that strengthen the sustainable goals, but not sufficient for exceeding the traditional growth-oriented model.

## 1. Introduction

Human wellbeing should represent the final objective of all endeavors carried out by humans. Consequently, satisfaction of the basic (economic, social, and environmental) needs of individuals becomes “the main societal objective” [1] (p. 220), [2] (p. 3), the center of all actions, at individual, group, community, regional, and/or national levels. Accordingly, the importance of the environment, of all debates around its degradation and of the acute necessity of protecting nature, appears as the result of the higher and higher awareness of the fact that human living, with all of its implications, is strongly affected by the environmental crisis [3,4,5]. In this regard, a considerable consensus on the fact that “we are facing it” is expressed [6]. Such concerns were not totally tangible in the past for the entire world population, but only punctually felt at specific local or regional levels. Contrarily, today, people from all over the world have to directly confront them as they are no longer theoretical ideas or isolated events, but concrete worriment asking for solutions. As Redclift and Benton [7] (p. 1) pointed out, the environmental concern was consolidated through the direct personal exposure to air pollution, congestion, and powerlessness regarding climatic anomalies, dietary worries, or other facets of “unwelcome development” related to nature. Even if the main reason for environmental degradation is linked to its under-evaluation and, consequently, to overuse or not, the problem has become one of the main issues of any discussion related to development. Thus, the environment no longer constitutes the “other”, it becomes an extension of the physical, living, cultural selves, networked in never-ending relations [6] (p. 110). Moreover, if considering our actual realities, it may possibly become the main objective.

Starting from this broad context, more and more specialists have pointed out that the extended alteration of the global environment was caused by human actions. This was made through their irrational (selfish) ways of attaining the objectives related to growth [8] (p. 11). Accordingly, stress was laid on quantitative issues, at the expense of qualitative ones such as types of goods and services to be provided, manner of production, and the ecological consequences of this process [7] (p. 3). On the other hand, analysis of the relationship between humankind and the natural environment is not a new subject. It represents one of the economic concerns already mentioned by classical and neoclassical economists (see Smith, Malthus, Ricardo, Mill, Jevons, Pigou, etc.), largely discussed by many authors over time [9,10,11]. As an overview, the literature related to development focuses either on economy, with its productive sectors, providing the incentives and means for both human wellbeing and for environmental maintenance and restoration [10,12,13], or on human development, with its major dimensions, such as life expectancy, education, and equity [13,14]. It has to be mentioned that both perspectives are closely engaged in environmental concerns. Regardless of the applied approach, as Wynne [15] (pp. 179–180) mentioned, it is justifiable to invest in environmental protection even before evidence of the cause–effect relationship is confirmed. This happens especially in the case in which it is reasonably anticipated that environmental discharge may be irreversibly harmful. Moreover, compliance with the requirements of environmental policy demands reduction in the environmental impact of industry, imposing fundamental changes in economic structures and processes, different from those practiced by conventional economics [16] (p. 5). In this context, Jacobs [17] is remembered by his remark on the essential character of production and consumption patterns common for this type of economics as the basis of the most stringent environmental problems [16] (p. 5).

As a response to the different obvious realities regarding the dimensions of sustainability, the adepts of ecological economics formulated a proposal for a new economic paradigm [11,18,19]. Its perspective is different from the conventional one, being intended to respond more properly to the desideratum of sustainable development, appearing as a new economic model focusing not on growth, but on wellbeing, especially the human one, as a main goal of economics. Accordingly, the center of gravity shifts from growth to the human quality of life, closely related to environmental peculiarities and protection. In detail, (1) development means sustainable progress of human wellbeing, while remaining fully aware that growth may produce negative effects, (2) individuals are responsible for understanding the peculiarities of the entire system and of the manner in which they have to sustainably act, (3) the objectives at micro levels need to be adjusted to reflect the main objectives of the system, and (4) focus is laid on the connections between people (society, with its two dimensions, i.e., social and economic) and the rest of the nature (i.e., environmental dimension) [11] (pp. 56–59).

### 1.1. Main Research Goals

We start from all these premises, as well as from the observation of Springett and Redclift [16] (p. 5), according to whom the environmental crisis was not a diversion from social ills, i.e., social problems or social issues, such as, for example, problems of social injustice, consequences of war, and the impacts of capitalism, but a result of them. Consequently, our research is intended to analyze the manner in which the effort of improving human wellbeing, next to economic wellbeing, contributes to the status of environmental wellbeing. This is not an easy question to answer; however, our study was aimed at capturing the indicative lines that delineate the reality, although it does not establish ultimate figures in this regard. Nevertheless, it is able to respond to the question regarding the manner in which human and economic wellbeing and their components influence environmental wellbeing: (1) if the former negatively influence the latter or (2) if the direction of such an influence changes as the development process becomes more sustainable-oriented in a certain context, i.e., EU countries. In our opinion, this is not a simple or meaningless approach, but one of high interest for the actual international context. We based our effort on the assertion of Collin and Collin [20] (p. 214), according to whom “environmental information moves sustainable development to implementation”. This perspective could represent one of the main reasons that potentially make our study of interest for the academic literature, as well as for the political, social, and/or economic spheres. In this respect, it firstly tries to analyze the manner in which the model of sustainable development is put into practice in the EU context. Secondly, on the basis of our study’s results, it opens new areas of research and policy initiatives that need to be fully comprehensive. Accordingly, we established our working hypotheses as follows:

**Hypothesis** **1.** *The levels of economic, social, and environmental dimensions significantly differed among the EU countries in 2019, being possible to observe a geographical grouping of different levels of sustainability.*

**Hypothesis** **2.** *The development patterns put into practice differed among the EU member states, such that the economic, social, and environmental dimensions of sustainability registered a different evolution in this group of countries across the 2006–2019 period.*

**Hypothesis** **3.** *The determinants of environmental wellbeing in terms of the components of economic and social dimensions of sustainability differed in the EU countries as a function of their national environmental status.*

A sustainable society is one that registers high levels of economic, social, and environmental wellbeing. Fully aware of the fact that nations differ in their capacity of attaining economic, social, and environmental performances (see, for example, Van de Kerk and Manuel [21]; Ulman et al. [22,23]), in detail, the first major aim of this paper was to compare the national levels between 2006 and 2019 in the EU member states, for establishing (1) national perspectives in terms of differences between the performances of the three sustainability dimensions, (2) similarities and differences among countries in this regard, and (3) evolution, from the perspective of time, of the three dimensions of sustainability within countries and also among them, between the two years of reference, i.e., 2006 and 2019. Consequently, we grouped the EU countries as a function of the economic, social, and environmental wellbeing recorded in 2006 and 2019. In this respect, we aimed to observe any possible changes at the level of each cluster’s components, as well as the nature of the migrations from one cluster to another, in terms of each sustainable dimension and of its components, while following the progress or regress observed across countries. Accordingly, this analysis permitted us to observe the strong and weak points of each cluster in terms of national capacity of assuring sustainability with regard to its main dimensions. In addition, we observed the nature of the influence of human and economic wellbeing upon environmental wellbeing in the groups of EU countries recording the lowest and the highest national environmental performance in 2019. In this way, we centered our debate on environmental wellbeing for finding out, on one hand, if countries follow different paths comparatively with the other two dimensions of wellbeing and, on the other hand, the nature of the influence of society’s performance (with its economic and social parts) upon it.

Consequently, we consider it especially important to have a general image of the national performances expected for acquiring (or not) high levels of sustainability, an aspect supported by the idea that sustainability is primarily “of the ensemble” [10] (p. 24). From this perspective, we see that, in the EU, there are still unanswered questions on the subject, an analysis able to offer a general image and to bring objectivity in terms of countries’ performances in this regard being legitimate and useful. In this way, considering the powerful causalities manifesting in the relations between society (with its two dimensions) and environment, some conclusions possibly omitted in all studies that analyzed sustainability in different ways would be both pertinent and useful. Thus, we sought to find out the resorts of environmental wellbeing from economic and social points of view, as well as the main weak points related to it in the EU countries. Accordingly, we intend to properly respond to the urgent call stated by Redclift and Benton [6] (p. 11), that of “putting social scientific ideas and methods to work in the understanding of our environmental crisis”. We started our endeavor with some critical questions on the capacity of nations to attain certain levels of sustainability, in terms of its main dimensions and subdimensions: (1) Are the EU member states close to being sustainable or is sustainability still a far-off objective in this group of countries? (2) What are the dimensions in which they perform better? (3) Do countries follow different paths in terms of the three dimensions of sustainability? (4) Is it possible to find out similarities among EU countries with regard to sustainability performances? (5) How do they manage to solve the trade-offs among the three dimensions for attaining a certain level of sustainability?

### 1.2. Conceptual Framework for Analyzing the Link among the Environmental, Social, and Economic Dimensions of Sustainable Development

Although adoption of the concept of sustainable development brought with it epistemological and practical problems, it also produced a transformation in the environmental discourse [16] (pp. 6–7). Thus, it encapsulated a more inclusive approach to living with nature and with each other [24] and, accordingly, the need for a radical change toward a different way of life, characterized by material simplicity and spiritual richness [25] (p. 132). As stated by many authors, sustainability is a multidimensional phenomenon, simultaneously including economic, social, and environmental dimensions. This peculiarity explains the complexity of the phenomenon, difficult to be captured within a single equation [10,26]. It represents one of the main causes of the fact that these three dimensions have not yet been reconciled as an organic whole [27]. The most common approach is to consider some (especially the three) dimensions of sustainability, the trade-offs between the objectives of each of them having to be present and continuous, until acceptable values for the society as a whole are reached [28,29].

Taking into account our main research goals and considering the dilemmatic character [30] of the human–nature relations, revealing diverse problematic aspects of the social and local contexts of action and amply discussed in the scientific literature related to environmental economics, special stress should be laid on the responsibility of humans, expected to better understand their role in relation to the environment [11] (p. 56). As a consequence of this major concern, the theory elaborated around this topic evolved and became specialized in different areas of environmental aspects, such as biodiversity [31,32,33], renewable water resources [34,35,36], consumption [8,37,38], energy use [39,40,41] and savings [42,43,44], greenhouse gases [45,46], or renewable energy [47,48,49] (see Table 1). Their integration was seen as translating into a unitary perspective on what we now call environmental wellbeing (see Van de Kerk and Manuel [21], as well as Kowalski and Veit [50]) (Table 1). Taking into consideration the fact that our study had as its basis the concept of wellbeing as defined by Van de Kerk and Manuel [21] in their Sustainable Society Index, we chose to refer here only to these main components integrated within this composite indicator. Their definitions are resumed in Table 1, also mentioning that there is an extensive literature analyzing each of them separately and in detail. Accordingly, some of the relevant studies are indicated in the same table. Considering that our approach is a general one, observing a phenomenon from a larger perspective, we opted to point out only the generic lines regarding each component of environmental wellbeing. The same rationale was used for the other two dimensions, i.e., social and economic ones, in addition to their relationship with environmental wellbeing, as found across the selected literature.

Human wellbeing is defined as formed by nine components (see Van de Kerk and Manuel [21], as well as Kowalski and Veit [50]), analyzed in the literature in relation to the environment, as follows: sufficient amount of food [51,52,53], sufficient to drink [54,55,56], safe sanitation [22,53,57], education [22,53,58,59], health [22,53,60,61,62], gender equality [21,23,63,64], income distribution [20,22,53,65,66,67], population growth [53,68,69,70,71], and good governance [22,53,59,72,73,74] (see Table 2). For example, it was demonstrated that (1) the lack of safe sanitation represents a pollutant factor, negatively influencing environmental wellbeing [22,57], (2) the level of individual education registers positive effects on pro-environmental attitudes [58,59,75], while the number of students enrolled in education may influence environmental wellbeing in a different manner, as a function of the analyzed stage of national development [22], (3) gender equality plays a role in environmental protection, but its influence is not unitarily understood [23,63,64,76], (4) less income inequality seems to translate into less environmental degradation [20,67], and (5) governance still has to improve its influence upon the environment, being possibly more oriented toward economic results [72,73,74].

In addition, economic wellbeing integrates another five components (see Van de Kerk and Manuel [21], as well as Kowalski and Veit [50]), also analyzed in the literature of the field in close relation with their influence upon the environment: organic farming [22,77,78,79,80,81], genuine savings [82,83,84], gross domestic product [22,85,86,87,88], employment [22,89,90], and public debt [22,91,92,93] (see Table 3). For example, (1) the contradictory findings regarding the benefits of organic farming upon the environment [22,78], (2) the theory of the U-shaped relationship between national income and environmental degradation, supported by the results of studies such as those of Ulman et al. [22] or Ergun and Rivas [88], (3) the expenses of growth, also including aspects other than GDP, for a satisfactory functioning of the economy–environment system [83], apparently harmful for the environment [22], (4) the potential conflict between environment and employment, as emphasized by Lawn [94], but not agreed on by other studies, considering unemployment as related to environmental taxes and, therefore, producing a lower financial support for its protection [89,90], and (5) public debt, representing a constraint for environmental quality [22,92,93], were pointed out across different studies.

The unitary approach in terms of environmental, human, and economic wellbeing is especially useful when we need a larger perspective over the society as a whole, for understanding it as a system in which everything relates to everything, and nothing can be understood without analyzing the entire system (see Lotka [11] and Costanza et al. [95]).

**Figure 1:**
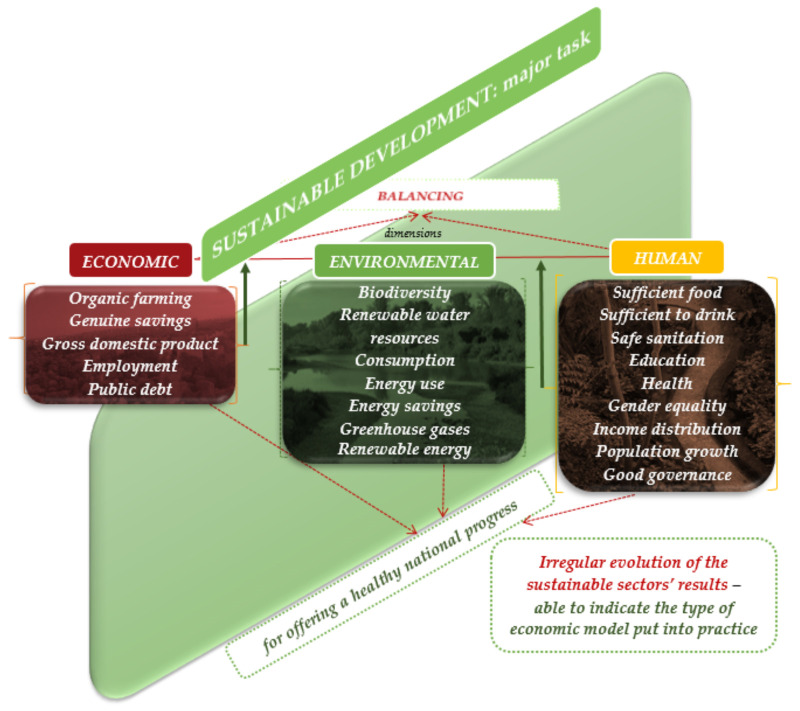
Conceptual framework for analyzing the relation among the environmental, social, and economic dimensions of sustainable development. Source: Authors’ representation following the approach of the SSI framework.

Therefore, sustainability appears as a necessary process of reconciliation and balance among different tendencies occurring in the three dimensions of sustainability (Figure 1). Our study might reveal if salient differences between the levels of each dimension are present across the investigated countries. Finding such differences of levels could offer the possibility to conclude on the type of model put into practice or the manner in which priority is granted, in a certain country, to one dimension or another. It might also be a way of investigating the manner in which economic and social sectors still influence the environmental quality, and whether differences appear in this regard among groups of countries with opposite levels of environmental wellbeing.

As far as we know, there are no other studies focusing on the human and economic components of wellbeing as determinants for environmental wellbeing in the European Union, analyzed through similar lenses, with the main aim of offering a general perspective to this aspect. An almost similar approach was followed by Ulman et al. [22], yet with a focus on the central and eastern European countries, as a group of nations that are currently in a developing process, with approximately similar national characteristics [22] (p. 6). In our opinion, this type of study is important because, as mentioned by Banerjee [96] (pp. 216–217), countries seem to vary enormously in their performance, standard macro-factors, macro-measures, policies, and institutional quality, such that national analyses are more appropriate. In addition, the European Union context is currently of great interest, yet not as homogeneous as it may appear, including countries that follow distinct paths and strategies of development.

The paper has four sections. After the introduction, Section 2 comprises a description of the data and used methodology. The results are presented and discussed in Section 3. The paper ends with conclusions in Section 4.

## 2. Data and Methods

Our analysis was focused on sustainable development across the European Union countries, using data collected between 2006 and 2019 from the official website of the Sustainable Society Index, which reports data every 2 years. Taking into account data availability, we extended the analysis over the entire period and, for the years with missing data (2007, 2009, 2011, 2013, 2015, and 2017), we calculated the mean values from the previous and subsequent years.

In the academic literature, many composite indices have been created in order to measure the sustainability of a country: the Environmental Performance Index (EPI) [97], the Human Development Index (HDI) [98], the Index of Sustainable Economic Welfare (ISEW) [99], the Sustainable Society Index (SSI) [21,50], the Ecological Footprint (EF) [100], etc. We choose to use SSI instead of other indices, taking into consideration three reasons. Firstly, the Sustainable Society Index comprises a large set of indicators for each of the three components of wellbeing (i.e., human wellbeing, economic wellbeing, and environmental wellbeing). Secondly, the indicators forming the framework of the index come from a large number of reliable sources (Table 4). Thirdly, the Sustainable Society Index was confirmed by the Joint Research Centre of the European Commission as an index “well-structured and guarantying a control process to ensure transparency and the credibility of the results” [101].

In addition, Witulski and Dias [102] stated that “the SSI conceptual framework can capture the overall picture of sustainability, and its modified measurement version that estimates the three dimensions simultaneously improves the convergence with well-known partial indices: the HDI for social and economic and the EPI for the environment component”. Moreover, Savić et al. [103] mentioned that “numerous sustainability composite indicators were developed and proposed in recent decades. Among all of them, only SSI approach tends to consider all three dimensions of sustainable development integrally”. In the same way, Luukkanen et al. [104] stated the fact that SSI “provides quite a comprehensive and reliable source of data”. Accordingly, we based our endeavor on the fact that the SSI is a reliable indicator and capable of intercepting the most important facets of sustainability.

The values of SSI indicators, categories, and dimensions have a variation range between 1 and 10. If a country is 100% sustainable in the case of an indicator, a category, or a dimension, its score is equal to 10. Otherwise, if a country is not sustainable at all for an indicator, a category, or a dimension, it would be scored with 1.

As already mentioned, our study was aimed at analyzing the environmental wellbeing in relation to human and economic wellbeing at the level of EU countries over the 2006–2019 period. Accordingly, we structured our analysis into two parts.

Firstly, we wanted to identify the changes produced in the evolution of the three dimensions and of their components. To this end, for each dimension, on the basis of the data presented in Appendix A, we performed two cluster analyses, one for 2006 and one for 2019. Using the two-step clustering method, we established three clusters as results of each cluster analysis: cluster 1, countries having low levels on the considered components; cluster 2, including countries with medium levels of components; cluster 3, including countries with high levels of the corresponding components.

Secondly, starting from the main results obtained in the cluster analysis performed in 2019 at the level of environmental dimension’s components, we identified significant differences among countries from cluster 1 and those from cluster 3. In order to understand the influence of the human and economic components on the environmental dimension in these two groups of countries over the entire period (2006–2019), panel data-specific methods were applied.

Panel data analysis was conducted in four steps. In the first one, we estimated three types of models: the pooled OLS (POLS) model, the fixed effects (FE) model, and the random effects (RE) model. In the second step, we applied several tests to identify the most suitable model for our data: Chow test for choosing between the POLS and FE model [105], Breush–Pagan test for choosing between the POLS and RE model [105], and Hausman test for choosing between the FE and RE model [106]. In the third step, for validating the selected model, we checked the following hypotheses: independence, homoscedasticity, autocorrelation, and multicollinearity. As not all these hypotheses were attained, in the last step, we estimated a corrected model. Taking into consideration that the number of years (14) was higher than the number of countries (nine in cluster 1 and three in cluster 3), we applied the feasible generalized standard squares (FGLS) model [107], thus obtaining our final results.

## 3. Results

### 3.1. Human Wellbeing

Analysis of countries’ grouping in terms of human wellbeing’s components, as well as the changes having appeared among them between 2006 and 2019 regarding their belonging to one group or another, evidenced three situations: (1) countries that have not changed their group, remaining on approximately the same path of wellbeing (Bulgaria, Finland, Greece, Romania, Sweden, etc.), (2) countries progressing to a group with better results of some components of human wellbeing (Austria, Estonia, Poland, Slovenia etc.), and (3) countries regressing to a lower-performing group (Ireland, Spain). This does not necessarily assume absolute lower levels of wellbeing, but different relative ones, resulting from the comparison made among countries and their different evolution across the analyzed period of time.

**Figure 2:**
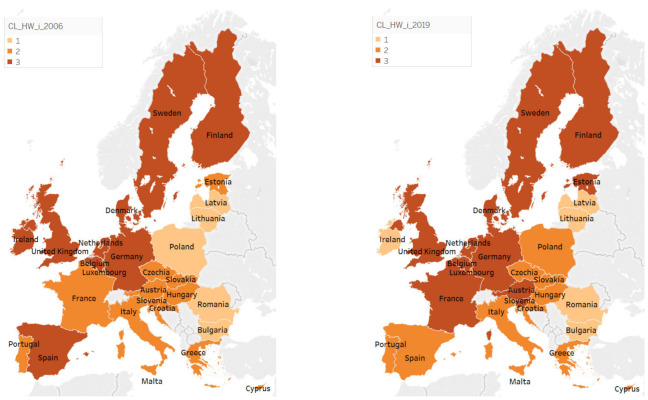
Clusters of human wellbeing components in 2006 vs. 2019. Source: SSI database, computed in Tableau Public v. 2021.3.

As shown in Figure 2, if the group of countries with the lowest levels of human wellbeing’s components included, in 2006, five countries, especially from the eastern part of Europe (Latvia, Lithuania, Poland, Romania, and Bulgaria), in 2019, their number remained the same; however, Poland was replaced by Ireland, which regressed from the cluster with the highest level of human wellbeing to the current one.

In the countries from this group, in 2006, the lowest levels of the components of human wellbeing were HW_GG (mean value of 5.84) and HW_ID (mean value of 6.280), with the highest levels being registered for HW_SF (mean value of 10) and HW_SD (mean value of 9.58). In 2019, the lowest and highest levels referred to the same components: HW_GG (mean value of 6.400), HW_ID (mean value of 4.460), HW_SF (mean value of 9.940), and HW_SD (mean value of 9.860). In 2006 and 2019, no important improvements in the medium levels of components were recorded in this cluster, which shows that, generally, human wellbeing did not register much progress across a part of Europe. Contrarily, regress could be observed, as in the case of HW_SF (from 10 to 9.940), HW_ID (from 6.280 to 4.460), and HW_PG (from 9.280 to 8.800). Nevertheless, improvements are to be mentioned, especially in terms of HW_HL (from 7.360 in 2006 to 9.400 in 2019).

In this way, although improvements may be observed across components such as good governance and income distribution, they appear not sufficient (1) for catching up the more evolved countries in this regard, as well as (2) for attaining a satisfactory level of human wellbeing in this group of countries. Moreover, in 2019, the main weakness of these countries was still related to the abovementioned components, i.e., good governance and income distribution, alongside gender equality, the same vulnerabilities recorded in 2006 (with the exception of HW_HL, which improved its level considerably across the analyzed period). In this way, the countries from eastern Europe (Figure 2), forming a compact group on the map drawn in 2019 (to which Ireland was added) have to focus on these issues for achieving an improved level of human wellbeing, following a sustainable path.

Out of the 14 countries belonging to the second cluster (Austria, Croatia, Cyprus, Czechia, Estonia, France, Greece, Hungary, Italy, Luxembourg, Malta, Portugal, Slovakia, and Slovenia), in 2006, five of them migrated to the third group (Austria, Estonia, France, Luxembourg, and Slovenia), seeming to improve their status of human wellbeing in 2019. In addition, two other countries entered this second group, one improving its levels of wellbeing (Poland), the other (Spain) coming from the best-positioned cluster in terms of human wellbeing. In 2006, the lowest levels of the components of human wellbeing were recorded for HW_GE (mean value of 6.707) and HW_ID (mean value of 6.579), while the highest levels were registered for HW_SF (mean value of 10) and HW_SD (mean value of 9.964). In 2019, the lowest and highest levels were recorded for the same components: HW_GG (mean value of 6.583), HW_ID (mean value of 6.192), HW_SF (mean value of 9.892), and HW_SD (mean value of 9.992). In 2006, the main weakness in terms of human wellbeing seemed to be related to income distribution (6.579), gender equality (6.707), good governance (7.050), and population growth (7.264). In 2019, these four components still represented concerns, namely, income distribution (6.192), good governance (6.583), gender equality (7.017), and population growth (7.083), while, as observed, the levels of three of them (income distribution, good governance, and population growth) were even reduced.

Cluster 3, considered to have the highest level of sustainability in terms of human wellbeing’s components, suffered a significant reconfiguration over the analyzed period. Initially (in 2006) formed by nine countries, especially from the north and northwest parts of Europe (Belgium, Denmark, Finland, Germany, Ireland, Netherlands, Spain, Sweden, and the United Kingdom), it became the most numerous in 2019, coming to include 11 members (Austria, Belgium, Denmark, Estonia, Finland, France, Germany, Netherlands, Slovenia, Sweden, and the United Kingdom), with seven countries remaining since 2006 in this group, while Spain and Ireland migrated to the second and first groups, respectively. In 2006, the lowest levels of the components of human wellbeing were HW_PG (mean value of 6.722) and HW_ID (mean value of 6.822), while the highest levels were registered for the HW_SF (mean value of 10) and HW_SD (mean value of 9.967) components. In 2019, the lowest and highest levels were recorded for the following components: HW_PG (mean value of 6.836), HW_SF (mean value of 10), and HW_SD (mean value of 10). Consequently, a larger part of the map drawn in 2019 became dark-colored, indicating a higher performance of the European countries in attaining a satisfactory level of human wellbeing for its citizens. Following the sustainable development objectives, all countries should strive to enter this group, recording the highest level of sustainability in terms of its social dimension. Improvement of human wellbeing’s components from 2006 to 2019 could be observed across this group of countries (for example, medium level of education, health, gender equality, and income distribution). Nevertheless, in 2019, the main vulnerabilities remained related to gender equality, income distribution, population growth, and good governance, even if not at levels below 6.836. All these components registered progress across the analyzed period, with the exception of good governance, which recorded a lower level in 2019 (mean value of 7.827), compared to 2006 (mean value of 8.189).

**Figure 3:**
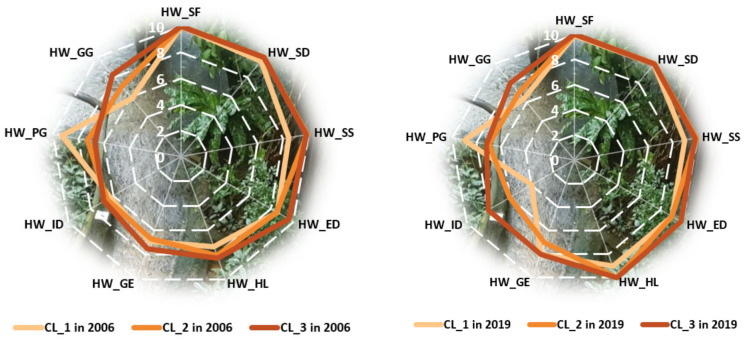
Comparison between clusters regarding human wellbeing components in 2006 vs. 2019. Source: SSI database, computed in Microsoft Excel 2013.

In the clusters formed in 2006 (Figure 3), similarities may be observed in terms of HW_SF, HW_SD, HW_SS (especially in the case of the last two clusters, with a lower level for the latter), HW_ED, HW_GE (especially for the first two clusters, with a higher level for the third one), and income distribution (with not very different levels, yet increasing from one cluster to another). A major difference can be observed for HW_GG, recording significantly increasing levels, as well as for HW_PG, with significantly decreasing levels from one cluster to another (from cluster 1 to cluster 3). As in 2006, HW_SF and HW_SD registered almost the same maximum levels as in 2019.

In addition, the HW_SS from the first cluster seemed to recover from the gap registered in 2006, compared with the other two clusters. Thus, although the countries from the group were disadvantaged in terms of human wellbeing, they improved their levels of safe sanitation, despite not attaining the same values as in the other clusters. Somehow, the gap between, on one hand, the first two clusters and, on the other, the last cluster regarding education was reduced, tending to be almost equal to zero in 2019. The major differences found in 2006 among the group of countries (in the case of good governance and population growth) were also maintained in 2019. An encouraging change regards income distribution, which increased its level in 2019 in the best-positioned group in terms of human wellbeing, thus being clearly delimitated from the groups registering lower levels.

### 3.2. Economic Wellbeing

**Figure 4:**
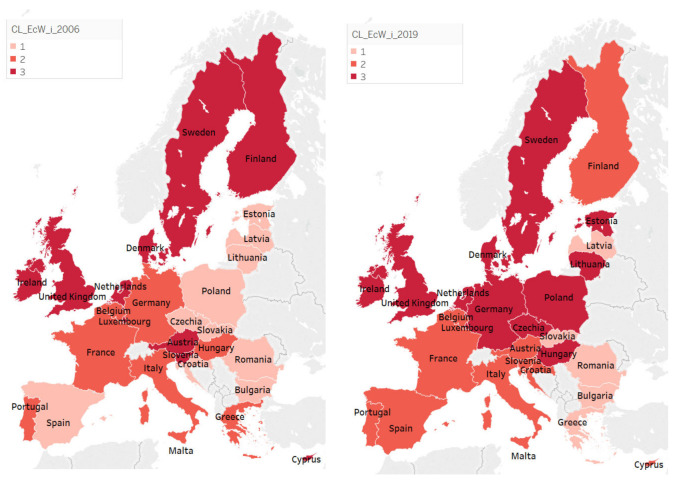
Representation of clusters regarding economic wellbeing components in 2006 vs. 2019. Source: SSI database, computed in Tableau Public v. 2021.3.

Analyzing countries’ grouping in terms of economic wellbeing’s components, as well as the changes manifested among countries from 2006 to 2019 with respect to their belonging to one group or another, three situations may be mentioned, similarly with the case of human wellbeing (Figure 4): (1) countries that have not changed their group, remaining on approximately the same path of wellbeing attainment (Denmark, Ireland, Sweden, UK, etc.), (2) countries progressing to a group with better results in terms of some components of economic wellbeing (Croatia, Estonia, Hungary, Poland, Spain, etc.), and (3) countries regressing to a lower-performing group (Austria, Finland, Greece, Slovenia).

In 2006, cluster 1 was formed by 10 countries, especially from eastern Europe (Bulgaria, Croatia, Czechia, Estonia, Latvia, Lithuania, Poland, Romania, and Slovakia), alongside Spain. A special characteristic of this group of countries is that, although its mean levels of the components of economic wellbeing were the lowest, compared to those of the other two groups of countries, they registered the highest level of wellbeing in terms of public debt (8.890, comparatively with 7.680 in the third cluster and 2.688 in the second one). In 2019, its constituents changed radically, as follows: Bulgaria, Greece, Latvia, Romania, and Slovakia. In this group, four countries, Romania, Bulgaria, Greece, and Slovakia, registered important economic progress due to their integration in EU. In addition, Greece progressed after the implementation of diverse restructuration programs as responses to its economic national crisis. In 2006, the lowest levels of the components of economic wellbeing were recorded for EcW_EMP (mean value of 3.150) and EcW_OF (mean value of 5.110), while the highest levels were registered especially for EcW_PD (mean value of 8.980). On the contrary, in 2019, the lowest levels were for the EcW_PD components (mean value of 6.140), alongside EcW_EMP (mean value of 4.260). Accordingly, as suggested by the obtained results, the change in terms of public debt was visible, shifting, in 2019, from a strong point (with a value equal to 8.980 compared to 2.688 and 7.680 for the other clusters) to the vulnerabilities of the countries from this cluster (despite recording the highest value among the three analyzed groups of countries). The highest level was registered for EcW_GDP (mean value of 8380), albeit not at a satisfactory level (in the context in which the other clusters registered mean values equal to 9.300 and 9.446). Thus, in 2006, the main weak points in terms of economic wellbeing seemed to be related to employment and organic farming, whereas, in 2019, the major concerns became public debt and employment.

These components appear to reflect major threats for sustainability in the situation in which public debts tend to increase more and more in the actual context, while chronic unemployment seems to be present in many of these analyzed countries.

A significant change was also registered, when analyzing economic wellbeing, in the second cluster. In 2006, it included Portugal, Italy, France, Greece, Hungary, Germany, Belgium, and Malta, whereas, in 2019, its members were Finland, Portugal, Spain, France, Belgium, Italy, Austria, Slovenia, Croatia, Cyprus, and Malta. In 2006, the lowest levels of the components of economic wellbeing were EcW_EMP (mean value of 4.400) and EcW_PD (mean value of 2688), while the highest levels were registered for EcW_GDP (mean value of 8.638) and EcW_GS (mean value of 8.050). In 2019, the lowest and highest levels were recorded for the following components: EcW_PD (mean value of 1.180), EcW_EMP (mean value of 3.980), EcW_GDP (mean value of 9.300), and EcW_OF (mean value of 8.770). In addition, the levels of wellbeing regarding the analyzed economic components tended to increase between 2006 and 2019, with the exception of public debt (from 2.688 in 2006 to 1.180 in 2019) and employment indicators (from 4.400 to 3.980), whose situation got worse, thus also remaining the main vulnerability of this group of countries in 2019.

In 2006, with reference to its economic components, cluster 3 was formed of Austria, Cyprus, Denmark, Finland, Ireland, Luxembourg, Netherlands, Slovenia, Sweden, and the United Kingdom, whereas, in 2019, Czechia, Denmark, Estonia, Germany, Hungary, Ireland, Lithuania, Luxembourg, Netherlands, Poland, Sweden, and the United Kingdom became part of it. In 2006, the highest levels of the components of economic wellbeing were EcW_GDP (mean value of 9.250) and EcW_GS (mean value of 8.850), with the lowest levels being registered for EcW_EMP (mean value of 5.800) and EcW_OF (mean value of 6.390). In 2019, the lowest and highest levels were recorded for the following components: EcW_EMP (mean value of 6.046), EcW_PD (mean value of 5.323), EcW_GDP (mean value of 9.446), and EcW_GS (mean value of 8.746). While the levels of wellbeing of organic farming, GDP, and employment improved between 2006 and 2019, those of genuine savings and public debt decreased. Although this group of countries seems to comprise the most economically favored nations, the decreasing level of sustainability in terms of public debt and genuine savings may become issues to be reflected upon. Nevertheless, the major concerns of these countries remain public debt and employment, which recorded a highly unsatisfactory level of wellbeing.

**Figure 5:**
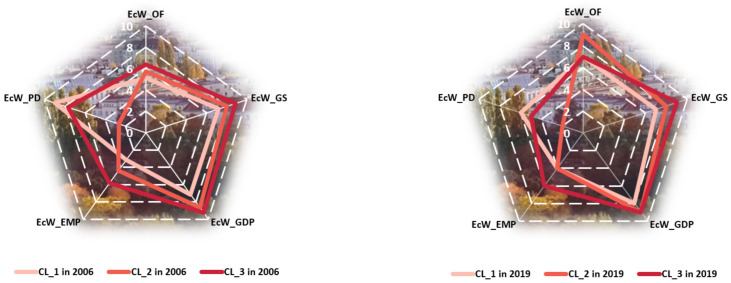
Comparison between clusters regarding economic wellbeing components in 2006 vs. 2019. Source: SSI database, computed in Microsoft Excel 2013.

Improvement of EcW_OF was registered especially in the first two clusters, while the third maintained almost the same level in 2019 compared to 2006. The EcW_GS parameter seemed to not improve its levels, remaining approximately similar for the analyzed groups of countries in 2019 and 2006 (Figure 5). The countries from the first cluster seemed to improve the level of wellbeing in terms of GDP, recovering some of the gap between it and the other two more economically sustainable clusters in 2006. In 2006, in clusters 2 and 3, employment seemed to register different levels, higher in the latter; in 2019, these groups of countries maintained their performance in this regard, with the second group coming to register almost a similar level of EcW_EMP as the first one.

Referring to public debt, major and negative changes were registered in the first cluster, i.e., the one with lower levels of economic wellbeing (from 8.980 in 2006 to 6.140 in 2019) and specific to the countries from central and eastern Europe. The countries from the last cluster decreased their levels of wellbeing, with the second still registering the most serious situation in terms of public debt.

### 3.3. Environmental Wellbeing

**Figure 6:**
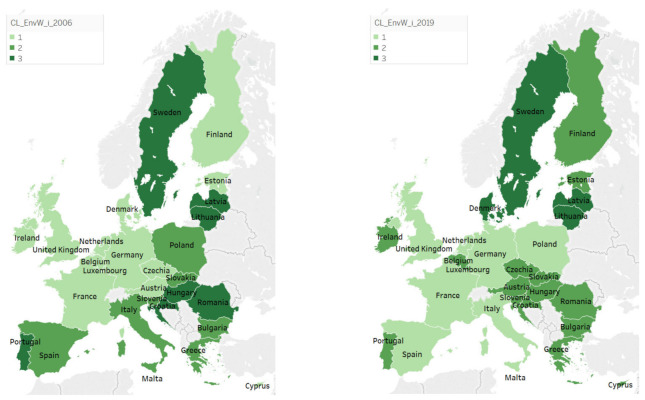
Representation of clusters regarding environmental wellbeing components in 2006 vs. 2019. Source: SSI database, computed in Tableau Public v. 2021.3.

Analyzing the following maps (Figure 6), greening of some nations may be observed in 2019 comparatively to 2006, in the context in which especially developed countries, such as Austria, Belgium, Ireland, Finland, and Denmark, showed improved environmental wellbeing. The contrary situation was also observed in some countries, such as Romania, Poland, and Croatia, as well as some more developed ones, such as Hungary, Italy, Portugal, or Spain, which took a step back in their status of environmental wellbeing. Nevertheless, the most common situation is that in which the countries maintained their affiliation to the same cluster, more frequently remaining, however, at a medium level of environmental wellbeing.

In 2006, cluster 1, characterized by low values of environmental wellbeing, included 13 countries, especially developed ones: Austria, Belgium, Cyprus, Czechia, Denmark, Estonia, Finland, France, Germany, Ireland, Luxembourg, Netherlands, and the United Kingdom. These are countries highly industrialized with permanent economic progress intensively affecting the quality of the environment. As a response, the European environmental policies were aimed at improving the most important environmental indicators. In 2019, it became less numerous, with only eight nations, France, Germany, Italy, Malta, Netherlands, Poland, Spain, and the United Kingdom, which indicates a trend of environmental wellbeing improvement across the member states of the European Union. This higher general environmental concern, materialized in higher levels of environmental quality, was manifested especially in developed countries such as Austria, Belgium, Denmark, Finland, and Ireland, which, in 2006, were shown to neglect environmental issues but, in time, using different strategies and paying closer attention to environmental protection, were able to somehow increase their environmental status. Returning to cluster 1, in 2006, the lowest levels of the components of environmental wellbeing were EnvW_GG (mean value of 1.262), EnvW_EU (mean value of 1.900), and Env_RE (mean value of 1.231), while the highest levels were registered for EnvW_RWR (mean value of 8.862) and EnvW_BD (mean value of 7.215). In 2019, the lowest and highest levels are recorded for the following components: EnvW_RE (mean value of 1.243), EnvW_ES (mean value of 1.286), EnvW_EU (mean value of 1.200), EnvW_RWR (mean value of 9.929), and EnvW_BD (mean value of 6.114). Unfortunately, compared with the levels of the other components of wellbeing (economic and human), in the case of the environmental ones, very low levels of wellbeing were observed. This finding highlights even more the necessity of focusing on the environment and on finding potential solutions for solving the environmental problems. In this context, the main vulnerabilities are related to renewable energy, greenhouse gases, energy savings, and energy use, still manifested during the 2006–2019 period, which recorded no significant improvement but, on the contrary, exhibited decreased energy use and savings.

In 2019, cluster 2, including countries with medium values of environmental wellbeing, became more numerous than in 2006, increasing from eight (Bulgaria, Greece, Italy, Malta, Poland, Slovakia, Slovenia, and Spain) to 15 countries (Austria, Belgium, Bulgaria, Croatia, Cyprus, Czechia, Estonia, Finland, Greece, Hungary, Ireland, Portugal, Romania, Slovakia, and Slovenia). Among them, only Bulgaria, Greece, Slovakia, and Slovenia remained in this cluster in 2019, whereas Italy, Malta, and Poland moved to the first cluster, and other countries, such as Austria, Belgium, Cyprus, Czechia, Estonia, and Ireland (with improved levels of wellbeing) or Portugal, Croatia, Hungary, and Romania (with lower levels) migrated to cluster 2. In 2006, the lowest levels of the components of environmental wellbeing from this cluster were recorded for EnvW_RE (mean value of 1.013) and EnvW_GG (mean value of 2.600), and the highest levels were registered for EnvW_BD (mean value of 8.275). In 2019, the lowest and highest levels were recorded for the following components: EnvW_RE (mean value of 1.550), EnvW_GG (mean value of 3.356), EnvW_CS (mean value of 3.800), EnvW_ES (mean value of 8.175), EnvW_EU (mean value of 8.119), and EnvW_RWR (mean value of 9.994). In this way, a significant positive change was observed for energy use and savings, with a considerably higher level of wellbeing, while renewable energy remained the main weakness of this cluster.

When analyzing the third cluster, the statistics showed that only three countries managed to maintain themselves in this group—with the highest values of environmental wellbeing—i.e., Latvia, Lithuania, and Sweden. Additionally, another developed nation from north Europe—Denmark—succeeded in improving its environmental quality. This weak improvement is not necessarily an unexpected one, as the developed countries were confronted with major environmental problems that required solutions for sustainable development, such that, along the years, different policies were put into practice, at both national and international levels, for attaining better environmental protection. Their effectiveness needs a thorough analysis, while important changes are required in this context, appearing as the most preferable in all Europe. Accordingly, the main vulnerability remains related to renewable resources, an essential strategic component of every nation, due to their significant benefits and unexhausted character, when considering that the fossil and nuclear fuels are running lower each day [48] (p. 78). In detail, in 2006, in this cluster, the lowest level of the components of environmental wellbeing was registered for EnvW_RE (mean value of 1.814), and the highest level was registered for Env_RWR (mean value of 9.486). In 2019, the lowest and highest levels were observed for the following components: EnvW_CS (mean value of 1), EnvW_RE (mean value of 2.5), EnvW_EU (mean value of 8.580), EnvW_ES (mean value of 8.620), and Env_RWR (mean value of 10). Such values again justify the concern for finding the most suitable solutions regarding consumption and renewable energy proposed in the literature (see Twidell [49]; Girardet and Mendonca [48]; Van de Kerk and Manuel [21]).

**Figure 7:**
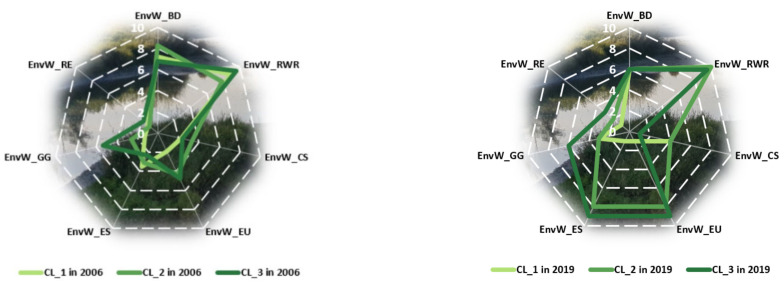
Comparison among clusters regarding environmental wellbeing components in 2006 vs. 2019. Source: SSI database, computed in Microsoft Excel 2013.

Comparing the status of environmental wellbeing for the two analyzed years, i.e., 2006 and 2019, an almost unchanged situation was met for renewable water resources and renewable energy (Figure 7). While one of them (renewable water resources) tended to register quite satisfactory level in terms of sustainability, the other (renewable energy) became a concern for environmental protection policy, once having registered very low levels of wellbeing, thus evidencing a critical situation compared both with the other environmental components of wellbeing and with the components of the other two dimensions of wellbeing, i.e., human and economic, registering the lowest level in the two analyzed years. Other major favorable changes were recorded in terms of energy savings and energy use across the countries from the last two groups, even if the first one was still characterized by very low levels. Moving on, the levels of wellbeing in terms of biodiversity significantly decreased between 2006 and 2019, registering approximately similar values for all three groups of countries under consideration. Accordingly, if in 2006, countries appeared to be more homogeneous in terms of environmental wellbeing and its components, in 2019, we faced an irregular image across Europe, with very different levels both among the groups of countries, on one hand, and among the environmental components, on the other. Unfortunately, as shown in the descriptive part of our analysis, only four countries managed to be included in the last cluster in terms of environmental wellbeing, i.e., Sweden, Denmark, Latvia, and Lithuania. Analyzed in detail, Sweden and Denmark belonged to the last clusters when economic and human wellbeing were considered, accordingly representing the best-performing countries in terms of sustainability across Europe. In addition, Lithuania failed to be integrated into the last cluster in terms of human wellbeing, whereas Latvia still remained in the first groups of countries in terms of both human and economic wellbeing.

These results clearly show that there is still sufficient room for improvement in terms of sustainability goals in the European Union and that the countries differ in their performance of attaining environmental, social, and economic wellbeing. Accordingly, our first hypothesis was confirmed. In addition, it was observed that the economic, social, and environmental dimensions of sustainability registered a different evolution along the 2006–2019 period in this context, confirming the second hypothesis of our study.

### 3.4. Environmental Wellbeing in Relation to Human Wellbeing

The influence of human wellbeing components on environmental wellbeing in the two clusters is presented in Table 5. In the countries from cluster 1 (i.e., France, Germany, Italy, Malta, Netherlands, Poland, Spain, and the United Kingdom), some of the human wellbeing indicators had a significant influence on environmental wellbeing (i.e., HW_SS, HW_HL, HW_PG, and HW_GG). In the countries from cluster 3 (i.e., Latvia, Lithuania, Sweden, and Denmark), most of the components of human wellbeing mentioned remained significant (i.e., HW_SS, HW_PG, and HW_GG), whereas HW_SS became insignificant and HW_SD became significant in relation to environmental wellbeing.

In the category of basic needs, two variables, HW_SD and HW_SS, were included, with variable HW_SF being omitted from both models because of collinearity. Regarding their influence on environmental wellbeing, it can be observed that only HW_SS had a significant and positive effect (Coef. = 2.198, Prob = 0.1) in the countries from cluster 1. Regarding cluster 3, both variables were significant and positive in relation to environmental wellbeing (Coef. = 2.298, Prob = 0.01 for HW_SD; Coef. = 2.162, Prob = 0.01 for HW_SS). Moving on to the second category of indicators (i.e., personal development and health), variable HW_HL was seen as having a significant and negative influence on environmental wellbeing in the model estimated at the level of cluster 1 (Coef. = −0.364, Prob = 0.01). In both models, variables HW_ED and HW_GE were found insignificant in explaining the variation of environmental wellbeing. In both models, variable HW_PG from the third category (i.e., wellbeing society) had significant and negative effects on environmental wellbeing (Coef. = −0.206, Prob = 0.01 for cluster 1 and Coef. = −0.355, Prob = 0.1 for cluster 3). Moreover, we can observe that, HW_GG had a significant and negative influence on the environmental wellbeing of the countries from both clusters (Coef. = −0.543, Prob = 0.01 for cluster 1 and Coef. = −2.148, Prob = 0.01 for cluster 3).

### 3.5. Environmental Wellbeing in Relation to Economic Wellbeing

The influence of economic wellbeing on environmental wellbeing in the two clusters is presented in Table 6. As seen, some differences may be observed between the influences of the considered factors on environmental wellbeing.

Starting from the first category of indicators referring to economic wellbeing (i.e., transition), we can observe a significant and negative effect of variable EcW_OF on the environmental wellbeing of countries from cluster 1 (Coef. = −0.071, Prob = 0.1), whereas, in the case of countries from cluster 3, the influence became positive (Coef. = 0.223, Prob = 0.1). The other variable from this category, EcW_GS, had a significant and negative influence only on the environmental wellbeing corresponding to the countries from cluster 1 (Coef. = −0.457, Prob = 0.01). Moving on to the second category of factors (i.e., economy), variable EcW_EMP had negative effects on the dependent variable in the countries from both clusters (Coef. = −0.190, Prob = 0.01 for cluster 1 and Coef. = −0.140, Prob = 0.1 for cluster 3). In addition, it is only in the model estimated for countries from cluster 3 that variable EcW_PD had a significant influence on environmental wellbeing (Coef. = −0.330, Prob = 0.05).

**Figure 8:**
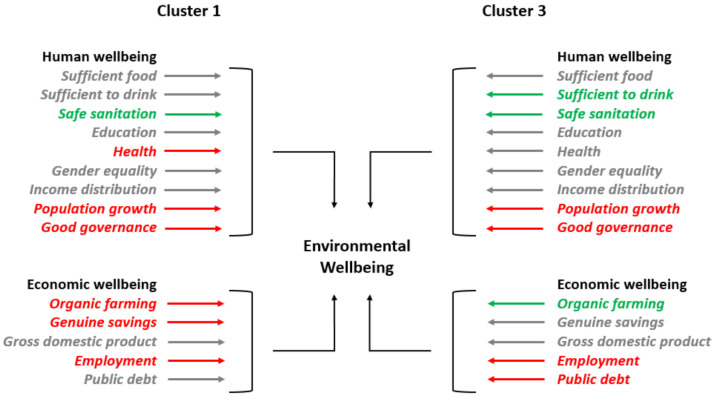
Comparison between clusters regarding the influence of human and economic wellbeing on environmental wellbeing. Source: Authors’ representation.

Figure 8 summarizes the results obtained in the panel data analysis conducted along the 2006–2019 period for each of the two clusters considered. As previously mentioned, cluster 1 included the countries with low levels of environmental wellbeing, while cluster 3 comprised the countries with high levels for this dimension of sustainability in the last year (i.e., 2019). For each of the two clusters, the influences of the factors in terms of human and economic wellbeing on environmental wellbeing were represented by differently colored arrows, i.e., gray for the factors with an insignificant influence on the dependent variable, green for a significant and positive influence, and red for a significant and negative influence.

Some human wellbeing indicators had the same influence on environmental wellbeing in both clusters (i.e., the positive influence of safe sanitation and the negative influence of population growth and good governance), while others changed their influence from a group to another (i.e., sufficient to drink from insignificant in cluster 1 to a significant and positive influence in cluster 3, health from a significant and negative influence in cluster 1 to insignificant in cluster 3).

Concerning the influence of economic wellbeing factors on environmental wellbeing, both similarities (i.e., employment had a significant and negative influence in both clusters) and differences could be observed (i.e., the genuine savings index had a significant and negative influence in cluster 1, while it was insignificant in cluster 3; organic farming had a significant and negative influence in cluster 1, but a significant and positive influence in cluster 3; public debt was insignificant in cluster 1 but had a significant and negative influence in cluster 3).

In this way, in the countries with the best environmental performance in Europe, such as Latvia, Lithuania, Denmark, and Sweden, factors related to basic needs, such as availability of drinking water in sufficient quantity and quality, and safe sanitation, or other factors related to a well-balanced society, such as population growth and good governance, tended to be significant in relation to environment. Among these factors, those related to basic needs, contrary to the others, succeeded in positively influencing environmental wellbeing. On the contrary, in countries with low environmental performance, such as Germany, France, Italy, Malta, Netherlands, Poland, Spain, and the United Kingdom, health negatively affected the indicator regarding the environment, whereas, in the other group of countries, this factor was found to be insignificant. In addition, population growth and good governance also appeared as negative factors in this group of countries, thus representing a vulnerability in terms of environmental wellbeing. In this way, firstly, in countries such as Germany, France, and Italy, following the path of the more environmentally oriented countries, issues such as health should be necessarily transformed from a factor negatively influencing the environment into a more ecofriendly one, capable of exercising a positive effect upon it, thus protecting and improving environmental wellbeing. Secondly, in addition to these weak points specific to the countries with low results in environmental protection, a common problem for both types of analyzed countries was the capacity of having a good governance at national level. This finding could reveal the incapacity of the governments in both categories of countries to sufficiently concentrate on the environmental dimension. Accordingly, the strategy of governing appears to be still predominantly traditional, oriented mainly toward economic results, and neglecting the necessity of a more efficient environmental protection. In the condition in which this phenomenon is present even in developed countries, even in those recording the best results in terms of environmental wellbeing, the developmental attitudes, actions, policies, and investments should be more carefully oriented toward solving the most stringent problems of environmental concern, such as consumption, energy use, energy savings, and greenhouse gases, while avoiding, through efficient public and private actions, the nonoptimal waste of national natural resources. Such an attitude should represent a moral and civic obligation, especially for the people involved in decision-making processes. This finding is consistent with that of Seifi [73], Stratan et al. [108], Ulman et al. [53], or Ulman et al. [22]. These authors also emphasized the role of good governance for avoiding environmental degradation, as well as the need for more pro-environmentally oriented policies.

In countries with better environmental performance, the environmental pressure seemed to be exercised by different types of economic factors. On one hand, such results seem to confirm the major concern pointed out in the study of Lawn [94], i.e., the potential conflict between environmental goals and employment, as also observed in the study of Ulman et al. [22], applied for the CEECs. On the other hand, they seem to emphasize that a high public debt most probably constitutes a significant constraint on environmental wellbeing, which confirms the findings of other studies, such as those of Ulman et al. [22], Fodha and Seegmuller [92], and Clootens [93], especially in the countries more pro-environmentally oriented. In addition, the fact that the genuine savings component was found to register a negative influence on the environment in the countries with weaker results on environmental wellbeing again emphasizes that a growing GDP is not sufficient for establishing whether national development follows a sustainable path. In this way, the growth expenses for maintaining the economy–environment system [83] could act as a harmful factor for the environment, especially, as already shown here, in the nations less oriented toward obtaining good environmental results. In addition, the contrary results obtained in the case of organic farming confirm the fact that the more pro-environmentally oriented countries succeeded in more efficiently implementing this type of farming, being shown to positively contribute to it.

As an overview, our empirical results reveal that the determinants of environmental wellbeing in terms of the components of economic and social dimensions of sustainability differ in the EU countries as a function of the national environmental status. This finding confirms our third hypothesis.

## 4. Conclusions

Satisfaction of the basic (economic, social, and environmental) needs of individuals represents the major societal concern, scope, and center of all particular, group, community, regional, and/or national actions, directed toward one or all dimensions of sustainable development.

A sustainable society is one that registers high levels of economic, social, and environmental wellbeing. Starting from the differences among nations with respect to their capacity of obtaining economic, social, and environmental performances, the major aim of the present paper was to compare the national levels, recorded between 2006 and 2019, of the EU member states, for providing (1) national perspectives in terms of differences among the performances of the three sustainability dimensions and of their components, (2) similarities and differences among countries in this regard, and (3) evolution of the three dimensions of sustainability within countries and among them, from the perspective of time, across the two years of reference, i.e., 2006 and 2019. Moreover, placing environmental wellbeing at the center of the debate, we intended to find out, on one hand, if countries follow different paths in this regard, compared to the other two dimensions of wellbeing and, on the other, the nature of the influence of society’s performance (with its economic and social aspects) upon it.

The general results obtained show that (1) there is still sufficient room for improvement in terms of sustainability goals in the European Union, (2) the economic, social, and environmental dimensions of sustainability registered a different evolution among the EU member states across the 2006–2019 period, with the development patterns put into practice differing in the European context, (3) the dimensions of sustainability were closely linked to each other along the analyzed countries, with both components of economic and social dimensions influencing the level of environmental wellbeing, and (4) the determinants of environmental wellbeing in terms of components of economic and social dimensions of sustainability differed in the EU countries as a function of their national environmental status.

In addition, considering that there are still unanswered questions on the subject in the EU context and reflecting on the strong causalities in the relation between society (with its two dimensions) and environment, we aimed to find out the resorts of environmental wellbeing from economic and social perspectives, as well as their main weak points. We concluded that the negative influences of economic and human components on the environment may represent specific directions of actions for alleviating them.

In detail, as an overview, firstly referring to human wellbeing across the EU member states, in countries such as Latvia, Lithuania, Romania, and Bulgaria, no important improvements in the medium levels of this dimension’s components were recorded from 2006 to 2019, which is a sign of the fact that, generally, human wellbeing has not registered much progress across this part of Europe. Moreover, in this group of countries, regress could be observed for sufficient food, income distribution, and population growth. However, fortunately, a larger part of the EU map of 2019 became dark-colored compared to 2006, indicating a higher performance of the European countries in obtaining a satisfactory level of human wellbeing for its citizens. An improvement of human wellbeing’s components from 2006 to 2019 could be observed across the group of countries with the most satisfactory performance in terms of social issues, e.g., Austria, Belgium, Denmark, Estonia, Finland, France, Germany, Netherlands, Slovenia, Sweden, and the United Kingdom (for example, in the case of a medium level of education, health, gender equality, and income distribution). Nevertheless, in 2019, the main vulnerabilities remained related to gender equality, income distribution, population growth, and good governance, even if not at very low levels.

Secondly, with regard to economic wellbeing, although, in 2006, the main weak points of the countries with the lowest economic performance in EU seemed to be related to employment and organic farming, in 2019, the major concerns became public debt and employment (in Bulgaria, Greece, Latvia, Romania, and Slovakia). Moreover, although the group of countries initially formed by Austria, Cyprus, Denmark, Finland, Ireland, Luxembourg, Netherlands, Slovenia, Sweden, and the United Kingdom, before comprising Czechia, Denmark, Estonia, Germany, Hungary, Ireland, Lithuania, Luxembourg, Netherlands, Poland, Sweden, and the United Kingdom in 2019 seemed to be the most economically favored one, the decreased levels of sustainability in terms of public debt and genuine savings could become issues to be reflected. Nevertheless, the major concern of these countries remains public debt and employment, which registered a highly unsatisfactory level of wellbeing.

Thirdly, greening of some nations could be observed in 2019 compared to 2006, in the context in which especially developed countries, such as Austria, Belgium, Ireland, Finland, or Denmark, improved their environmental wellbeing. A different situation was met in countries such as Romania, Poland, and Croatia, as well as in some more developed ones, such as Hungary, Italy, Portugal, or Spain, whose status of environmental wellbeing regressed. Nevertheless, the most commune situation refers to the maintenance of nations belonging to the same cluster, the most numerous being those with a medium level of environmental wellbeing. Unfortunately, compared with the levels of wellbeing specific to the other economic and human components, very low levels were observed for the environmental ones. This finding strengthens the necessity of putting the environment into the center of the discussion, with all its concerns and potential solutions for finding responses to all environmental problems.

Lastly, the present study was aimed at better understanding the manner in which countries with better environmental performance manage to offer specific levels of human and economic wellbeing, while comparing their sustainable development pattern to that of the countries with lower environmental wellbeing. Accordingly, our analysis on the relation between, on one hand, the levels of environmental wellbeing and, on the other, the levels of the economic and human components, laid stress on some particular results. Thus, it was found that, firstly, for the countries with a better environmental performance, the components of human wellbeing, e.g., availability of drinking water, safe sanitation, population growth, and good governance, were significant; in the countries with lowest levels of environmental wellbeing, safe sanitation, health, population growth, and good governance were observed to be significant, whereas the remaining components were irrelevant. Secondly, in both groups of countries, organic farming and employment appeared to be significant; nevertheless, differences were observed for organic farming, for which the different patterns of economic activities seemed to be materialized as different influences upon environmental wellbeing. In this way, in the group of countries with low environmental performance, the main economic and social vulnerabilities seemed to be health, population growth, good governance, organic farming, genuine savings, and employment sectors, whereas, in the better-performing countries with respect to environment and its quality, the main vulnerabilities were related to population growth, good governance, employment, and public debt.

To conclude, our study draws attention to the fact that the patterns of development applied in the European context seem to have results capable of strengthening the sustainable goals, but not sufficient for proving that the traditional growth-oriented model has been exceeded or that a path has been drawn to a new economic development one. Focusing on the environment, this observation could be deduced from the fact that environmental wellbeing still registered low levels among the EU countries compared to the other levels of human and economic wellbeing, as well as from the negative influence of economic and social components still exercised upon environmental wellbeing in the analyzed context and period of time. Therefore, there is clearly room for improvement in the process of development directed to sustainability goals, through the factors still negatively linked to the wellbeing of our environment.

The evolution of the indicators in the analyzed period revealed profound changes specific to the European countries. These modifications were consequences of different contextual events such as the economic crisis, the integration of some countries into the EU, and some environmental/economic/social policies. As observed across the study, the image of the analyzed countries’ evolution seems to be diverse, with their performances tending to be oriented more or less to each of the sustainable dimensions.

Our research results should, however, take into consideration some limits. In this respect, a weak point of the study comes from the fact that we were not able to develop the analysis over a larger period of time and to use timeseries-specific methods, capable of offering a more accurate image of the analyzed phenomenon, because of a lack of data availability. In addition, the fact that the levels of each component of SSI are available once every 2 years could represent another limitation in terms of data. To address these shortcomings, in terms of checking and comparing the obtained results within this study, it is possible to employ a similar approach, but using different indices that evaluate the same issues, such as the Index for Sustainable Economic Welfare (ISEW), Environmental Performance Index (EPI), or Ecological Footprint (EF). This may represent potential future research. In addition, taking into consideration the fact that, in our study, we focused on the EU countries, future research should employ extended analyses at the regional and local levels in different countries. Furthermore, starting from our results, especially regarding the specific human and economic vulnerabilities identified in this study, future research may conduct deeper analyses in order to improve their thorough understanding, as well as observe the different ways in which their negative effects on environment could be alleviated.

## Figures and Tables

**Table 1:** Definition of environmental wellbeing’s components.

Environmental Wellbeing Components	Definition of the Used Indicator [21,50]	Relevant Literature
Biodiversity (EnvW_BD)	The 10 year change of forest areas and the size of protected land areas as a % of the total land area of a country.	[31,32,33]
Renewable water resources (EnvW_RWR)	Annual water consumption as a % of the total available renewable water resources, including internal and external (flowing in from neighbor countries) water resources.	[34,35,36]
Consumption (EnvW_CS)	Ecological footprint minus carbon footprint, once it is already included in this index by the emission of greenhouse gases.	[8,37,38]
Energy use (EnvW_EU)	Primary energy usage − production + imports - exports ± stock changes.	[39,40,41]
Energy savings (EnvW_ES)	Change in primary energy usage between 2012 and 2016, as a %.	[42,43,44]
Greenhouse gases (EnvW_GG)	Amount of emitted CO_2_—other GHG emissions not included, such as CH_4_, N_2_O, HFCs, PFCs, and SF6.	[45,46]
Renewable energy (EnvW_RE)	Share of energy produced by renewable sources, as a % of the total energy (TPES, total primary energy supply).	[47,48,49]

**Table 2:** Definition of human wellbeing’s components.

Human Wellbeing Components	Definition of the Used Indicator [21,50]	Relevant Literature—The Relation between Specific Human Indicator and Environment
Sufficient food (HW_SF)	Availability of at least the minimum level of dietary energy for each person—prevalence of undernourishment (%).	[51,52,53]
Sufficient to drink (HW_SD)	% of population using at least basic drinking water services.	[54,55,56]
Safe sanitation (HW_SS)	% of population using at least basic sanitation services.	[22,53,57]
Education (HW_ED)	% of combined gross enrollment ratio, primary to tertiary, both sexes.	[22,23,58,59]
Healthy life (HW_HL)	Life expectancy at birth as number of healthy life years.	[22,53,60,61,62]
Gender equality (HW_GE)	Gender gap index—based on 14 indicators aggregated into 4 categories: economic participation and opportunity; educational attainment; political empowerment; health and survival.	[21,23,63,64]
Income distribution (HW_ID)	Ratio of income share held by lowest 10% to income share held by highest 10%.	[20,22,53,65,66,67]
Population growth (HW_PG)	Average yearly 5 year change in population, total—a negative population growth.	[53,68,69,70,71]
Good governance (HW_GG)	Sum of the values of the six worldwide governance indicators—voice and accountability, political stability, government effectiveness, regulatory quality, rule of law, and control of corruption.	[22,53,59,72,73,74]

**Table 3:** Definition of economic wellbeing’s components.

Economic Wellbeing Components	Definition of the Used Indicator [21,50]	Relevant Literature—The Relation between Specific Economic Indicator and Environment
Organic farming (EcW_OF)	The area of fully converted and in-conversion organically cultivated land as percentage of the total agricultural area.	[22,77,78,79,80,81]
Genuine savings (EcW_GS)	The true rate of savings in an economy after taking into account investments in human capital, depletion of natural resources, and damage caused by pollution.	[82,83,84]
Gross domestic product (EcW_GDP)	The market value of all goods and services produced within a country in a given period.	[22,85,86,87,88]
Employment (EcW_EMP)	Unemployment, total (% of total labor force).	[22,89,90]
Public debt (EcW_PD)	General government liabilities or debt + loans or net lending.	[22,91,92,93]

**Table 4:** The SSI framework.

Dimensions	Human			Economic		Environmental	
**Categories**	**Basic Needs**	**Personal Development and Health**	**Well-Balanced Society**	**Transition**	**Economy**	**Natural Resources**	**Climate and Energy**
Indicators (Source)	Sufficient food (FAO FSI)	Education (UNESCO)	Income distribution (WB)	Organic farming (FIBL)	Gross domestic product (IMF)	Biodiversity (Protected Planet)	Energy use (IEA)
	Sufficient to drink (FAO FSI)	Healthy life (WHO HALE)	Population growth (WB)	Genuine saving (WB)	Employment (WB)	Renewable water resources (FAO Aquastat)	Energy savings (IEA)
	Safe sanitation (FAO FSI)	Gender equality (WEF)	Good governance (WB)		Public debt (IMF)	Consumption (GFN)	Greenhouse gases (IEA)
							Renewable energy (IEA)

Source: Authors’ representation following the SSI framework.

**Table 5:** Relation between environmental wellbeing and the components of human wellbeing in clusters 1 and 3.

Component			Dependent Variable: Environmental Wellbeing	
			Cluster 1	Cluster 3
Human wellbeing	Basic needs	HW_SF	(omitted) ^a^	(omitted)
		HW_SD	−3.249 (3.209) ^b^	2.298 *** (0.660)
		HW_SS	2.198 * (1.188)	2.162 *** (0.629)
	Personal development and health	HW_ED	−0.096 (0.190)	−0.228 (0.266)
		HW_HL	−0.364 *** (0.128)	0.136 (0.179)
		HW_GE	0.239 (0.333)	−0.292 (0.461)
	Well-balanced society	HW_ID	0.065 (0.050)	−0.042 (0.053)
		HW_PG	−0.206 *** (0.079)	−0.355 * (0.186)
		HW_GG	−0.543 *** (0.153)	−2.148 *** (0.489)
		(Constant)	21.648 (20.900)	−16.336 ** (6.903)
		Wald ${𝓧}^{2}$	45.42 *** (0.000) ^c^	102.13 *** (0.000)

Notes: ^a^ the variable was omitted because of collinearity; ^b^ denotes the standard error specific to each coefficient from the FGLS regression models; ^c^ denotes the probability of the Wald test. ***, **, and * denote statistical significance at the 1%, 5%, and 10% levels. Source: SSI database, computed in STATA v.13.

**Table 6:** Relations between environmental wellbeing and the components of economic wellbeing in clusters 1 and 3.

Component			Dependent Variable: Environmental Wellbeing	
			Cluster 1	Cluster 3
Economic wellbeing	Transition	EcW_OF	−0.071 * (0.039) ^a^	0.223 * (0.123)
		EcW_GS	−0.457 *** (0.128)	−0.003 (0.091)
	Economy	EcW_GDP	0.125 (0.196)	−0.237 (0.170)
		EcW_EMP	−0.190 *** (0.064)	−0.140 * (0.079)
		EcW_PD	−0.010 (0.044)	−0.330 ** (0.139)
		(Constant)	7.528 *** (1.464)	7.553 *** (2.221)
		Wald ${𝓧}^{2}$	38.52 *** (0.000) ^b^	31.70 *** (0.000)

Notes: ^a^ denotes the standard error specific to each coefficient from the FGLS regression models; ^b^ denotes the probability of the Wald test. ***, **, and * denote statistical significance at the 1%, 5%, and 10% levels. Source: SSI database, computed in STATA v.13.

## Appendix A

**Table A1:** Levels of human, economic, and environmental wellbeing in the European Union countries in 2006 and 2019.

Country	Human Wellbeing		Economic Wellbeing		Environmental Wellbeing	
	2006	2019	2006	2019	2006	2019
Austria	8.5	8.5	6.9	5.5	3.0	4.7
Belgium	8.4	8.8	4.3	5.1	2.3	3.7
Bulgaria	7.7	7.5	4.1	7.1	4.2	5.1
Croatia	8.1	8.4	4.6	4.6	4.3	6.4
Cyprus	7.8	8.3	6.0	4.5	3.2	5.1
Czech Republic	8.3	8.8	7.3	8.6	2.1	3.7
Denmark	8.9	8.9	8.0	8.0	2.8	4.8
Estonia	8.2	8.8	7.3	8.5	2.1	3.5
Finland	8.9	9.1	7.5	6.2	2.6	4.7
France	8.3	8.6	5.5	4.8	3.0	2.7
Germany	8.4	8.9	5.8	7.2	2.9	2.5
Greece	8.0	8.3	4.3	3.0	3.1	5.2
Hungary	8.3	8.3	5.8	6.0	3.9	5.4
Ireland	7.8	8.7	6.8	5.4	2.7	3.7
Italy	7.8	7.8	5.0	4.7	3.4	2.9
Latvia	8.0	8.3	6.5	7.1	3.8	4.6
Lithuania	8.1	8.2	6.3	7.6	3.0	4.9
Luxembourg	8.1	7.0	7.8	8.0	1.9	3.3
Malta	6.5	7.5	4.3	5.0	2.8	5.7
Netherlands	8.5	8.9	7.3	6.6	2.5	2.4
Poland	7.9	8.6	4.4	6.2	3.9	2.8
Portugal	7.8	8.7	6.3	4.7	3.9	4.6
Romania	7.7	7.5	4.4	6.6	3.9	5.0
Slovak Republic	8.2	8.4	5.5	6.8	3.9	5.3
Slovenia	8.4	9.1	7.6	7.8	3.4	4.8
Spain	7.8	8.4	6.6	4.2	3.1	3.0
Sweden	8.9	8.7	7.8	7.5	3.1	4.5
United Kingdom	8.1	8.8	7.3	4.8	3.2	2.8
